# A New Pathway for Mannitol Metabolism in Yeasts Suggests a Link to the Evolution of Alcoholic Fermentation

**DOI:** 10.3389/fmicb.2019.02510

**Published:** 2019-11-01

**Authors:** Carla Gonçalves, Carolina Ferreira, Luís G. Gonçalves, David L. Turner, Maria José Leandro, Madalena Salema-Oom, Helena Santos, Paula Gonçalves

**Affiliations:** ^1^UCIBIO-REQUIMTE, Departamento de Ciências da Vida, Faculdade de Ciências e Tecnologia, Universidade Nova de Lisboa, Caparica, Portugal; ^2^Instituto de Tecnologia Química e Biológica António Xavier, Universidade Nova de Lisboa, Oeiras, Portugal; ^3^Centro de Investigação Interdisciplinar Egas Moniz (CiiEM), Instituto Universitário Egas Moniz, Caparica, Portugal

**Keywords:** alcoholic fermentation, yeast metabolism, mannitol metabolism, *Starmerella*, fructophily

## Abstract

The yeasts belonging to the *Wickerhamiella* and *Starmerella* genera (W/S clade) share a distinctive evolutionary history marked by loss and subsequent reinstatement of alcoholic fermentation mediated by horizontal gene transfer events. Species in this clade also share unusual features of metabolism, namely the preference for fructose over glucose as carbon source, a rare trait known as fructophily. Here we show that fructose may be the preferred sugar in W/S-clade species because, unlike glucose, it can be converted directly to mannitol in a reaction with impact on redox balance. According to our results, mannitol is excreted to the growth medium in appreciable amounts along with other fermentation products such as glycerol and ethanol but unlike the latter metabolites mannitol production increases with temperature. We used comparative genomics to find genes involved in mannitol metabolism and established the mannitol biosynthesis pathway in W/S-clade species *Starmerella bombicola* using molecular genetics tools. Surprisingly, mannitol production seems to be so important that *St. bombicola* (and other W/S-clade species) deploys a novel pathway to mediate the conversion of glucose to fructose, thereby allowing cells to produce mannitol even when glucose is the sole carbon source. Using targeted mutations and ^13^C-labeled glucose followed by NMR analysis of end-products, we showed that the novel mannitol biosynthesis pathway involves fructose-6-phosphate as an intermediate, implying a key role for a yet unknown fructose-6-P phosphatase. We hypothesize that mannitol production contributed to mitigate the negative effects on redox balance of the ancient loss of alcoholic fermentation in the W/S clade. Presently, mannitol also seems to play a role in stress protection.

## Introduction

Fungi are excellent models in which to explore the evolution of metabolism, as shown by multiple studies focused chiefly on secondary metabolite production by filamentous fungi (Spatafora and Bushley, 2015; Wisecaver and Rokas, 2015; Theobald et al., 2018; Keller, 2019). Ascomycetous yeasts (Saccharomycotina) produce very few secondary metabolites and are mainly known for their ability to convert sugars into ethanol, a trait of wide biotechnological relevance. However, central carbon and nitrogen metabolisms are in fact quite diverse in yeasts, accounting for the utilization of very different carbon and nitrogen sources and different regulation of energy production processes (Kurtzman et al., 2011). Comparative genomics of a large and ever-increasing number of yeast genomes is beginning to shed light on the evolution of metabolic traits in this sub-phylum, thus far revealing a general trend of loss of metabolic capabilities that implies a common ancestor metabolically more complex than most extant species (Shen et al., 2018). These losses most often resulted in narrowing spectra of nutrient assimilation, but we reported recently that even a central metabolic feature like alcoholic fermentation, which usually plays a crucial role in the metabolism of yeasts, either as the main pathway for energy production or as a means to circumvent oxygen shortage, was lost in a lineage within the Saccharomycotina currently comprising approximately 100 species belonging to the genera *Wickerhamiella* and *Starmerella* (referred to as the W/S clade) (Gonçalves et al., 2018). An ancestor of this clade seems to have lost the genes required for alcoholic fermentation, encoding pyruvate decarboxylase and alcohol dehydrogenase, which are also absent in at least one extant species, *Candida* (*Wickerhamiella*) *galacta* (Gonçalves et al., 2018). Reinstatement of alcoholic fermentation was subsequently made possible through horizontal acquisition of a bacterial alcohol dehydrogenase, and co-option of a new decarboxylase to accept pyruvate as a substrate (Gonçalves et al., 2018). Alcohol dehydrogenase genes were among the more than 500 bacterial genes detected in W/S-clade genomes. Although this remains to be empirically demonstrated, the unusually high number of horizontally acquired genes in the W/S clade suggests that other aspects of metabolism were also impacted during evolution of the lineage (Gonçalves et al., 2018; Shen et al., 2018; Kominek et al., 2019).

One additional unusual trait in common between W/S-clade species is their preference for fructose over glucose when both carbon sources are present at high concentrations, a trait named fructophily. The preference for fructose relies on a highly unusual specific fructose transporter, Ffz1 (Pina et al., 2004; Leandro et al., 2011, 2014), present only in fructophilic yeasts, which does not belong to the sugar transporter family and was horizontally acquired from filamentous fungi by a W/S-clade ancestor (Gonçalves et al., 2016). Ffz1, like fructophily, is present in most W/S-clade species examined and was shown to be indispensable for preferred fructose metabolism in at least one species in this clade (Gonçalves et al., 2018). However, while Ffz1 ensures that cells can take up large amounts of fructose in a selective manner, it remained unclear why the preferential utilization of fructose was beneficial for W/S-clade yeasts. Most species included in the *Wickerhamiella* and *Starmerella* genera have an ecological association with the floral niche, being often isolated from flowers or insects that visit flowers (Lachance et al., 2001; Canto et al., 2017; de Vega et al., 2017). In floral nectars, overall sugar concentrations are very high and fructose is usually roughly as abundant as glucose, sucrose being frequently the main sugar present (Canto et al., 2017; de Vega et al., 2017). Why, in this kind of environment, fructose assimilation should be more advantageous than that of other equally available sugars remained unknown. However, the metabolism of fructophilic bacteria that are also associated with the floral niche (Endo et al., 2009, 2010; Neveling et al., 2012) provides a useful clue. In fructophilic bacteria, the loss of alcoholic fermentation appeared to be linked to acquisition of fructophily because fructose can function both as carbon source and as an electron acceptor (in a reaction that converts fructose to mannitol), ensuring redox balance in the absence of alcoholic fermentation (Maeno et al., 2016, 2019). Interestingly, some fructophilic yeasts from the W/S clade are also known to produce large amounts of mannitol (Savergave et al., 2013), suggesting a possible link between mannitol metabolism and the evolutionary history of alcoholic fermentation in this lineage. On the other hand, mannitol often plays stress protective roles, namely as compatible solute (Chaturvedi et al., 1996; Solomon et al., 2007; Wyatt et al., 2014; Zahid and Deppenmeier, 2016), and W/S-clade yeasts are known to be osmotolerant (Rosa and Lachance, 1998).

To shed light on the role played by mannitol in the metabolism of W/S-clade yeasts, we used comparative genomics and metabolic characterization of mutants constructed in the genetically amenable W/S-clade species *Starmerella bombicola.*

Our findings suggest that mannitol metabolism was important to the fulfillment of redox balance in the absence of alcoholic fermentation, because direct conversion of fructose into mannitol regenerates NADP^+^. In support of this, we uncovered a novel biochemical pathway promoting the conversion of glucose to fructose. This pathway, which to our knowledge has not been reported before, enables mannitol production even when glucose is the only sugar available. Hence, we unraveled further the biochemical basis for fructophily in these yeasts and present a model for the evolution of metabolism in the W/S clade in which fructophily is linked to mannitol production and possibly to the ancient loss of alcoholic fermentation in this lineage.

## Materials and Methods

### Yeast Strains and Culture Conditions

*St. bombicola* PYCC 5882, *Candida magnoliae* PYCC 2903, *Starmerella bacillaris* PYCC 3044, *St. bacillaris* PYCC 6282 and *Wickerhamiella domercqiae* PYCC 3067 were obtained from the Portuguese Yeast Culture Collection (PYCC). *Wickerhamiella versatilis* JCM 5958 was obtained from the Division of Genomic Technologies, RIKEN Center for Life Science Technologies, Japan. All strains were maintained in YPD medium [1% (w/v) yeast extract, 2% (w/v) peptone and 2% (w/v) glucose)].

### Assessment of Mannitol Production in W/S-Clade Species

W/S-clade species (*St. bombicola*, *St. bacillaris*, *C. magnoliae, W. domercqiae* and *W. versatilis*) were cultivated in rich medium [YP, 1% (w/v) yeast extract and 2% (w/v) peptone] supplemented with 10% (w/v) glucose or 10% (w/v) fructose at 30°C until late exponential phase (when most of the sugar was consumed). Extracellular concentrations of fructose, glucose and other extracellular metabolites including mannitol were determined by HPLC using a carbohydrate analysis column (300 mm × 7.8 mm, Aminex HPX-87P, Biorad) and a differential refractometer (LKB 2142). The column was kept at 80°C and H_2_O was used as the mobile phase at 0.6 mL min^–1^. The results were treated and integrated using the software Chromeleon v6.8 (Dionex).

### Identification of Genes Involved in Mannitol Biosynthesis in W/S Genomes and Construction of Phylogenies

Genes possibly involved in mannitol biosynthesis, namely mannitol dehydrogenase (*MtDH*), mannitol-1P dehydrogenase (*M1PDH*) and mannitol-1P phosphatase (*MPP*), were searched in W/S-clade genomes by tBLASTx, using as query *MtDH* from *Yarrowia lipolytica* (*YlSDR*, accession number: Q6CEE9.1), *M1PDH* from *Aspergillus fumigatus* (KEY81154.1) and *MPP* from *Saccharina japonica* (AIC75600.1). An *E*-value cutoff of e^–10^ was considered for the tBLASTx search. The best hits were used as queries for BLASTp against the NCBI nr database to identify the closest related protein sequences in other yeasts (Saccharomycotina). No hits were obtained for genes other than *MtDH* and for that reason a detailed Maximum Likelihood (ML) phylogeny was constructed for the protein encoded by this gene. To that end, the top 750 hits BLASTp hits using the Mtdh-like protein from *St. bombicola* in UniprotKB (reference proteomes database) were selected to construct a ML phylogeny. Mtdh sequences from other W/S-clade species were retrieved by tBLASTx searches in local genome databases. The resulting protein sequences were aligned using an iterative refinement method (L-INS-i) in *MAFFT* v.6.956 (Katoh and Standley, 2014) and poorly aligned regions were removed with the *trimAl* (Capella-Gutierrez et al., 2009) software using the ‘gappyout’ option. An ML phylogeny was constructed with *IQ-TREE* v 1.4.3 (Nguyen et al., 2015) using the ultra-fast bootstrap method and the LG + I + G model of substitution (found as the best-fitting model). Accession numbers for W/S-clade genomes are the following: GCA_003033765.1 (*St. bacillaris* PYCC 3044), GCA_001005415.1 (*Candida apicola* NRRL Y-50540), GCA_003033435.1 (*St. bombicola* PYCC 5882), GCA_003033705.1 (C. *magnoliae* PYCC 2903), GCA_001600375.1 (*W. domercqiae* PYCC 3067), GCA_001005415.1 (*W. versatilis* JCM 5958) and NW_020193984.1 (*Wickerhamiella sorbophila* DS02). Complete phylogeny and alignment files can be accessed in figshare (10.6084/m9.figshare.9959693).

### Construction of *Starmerella bombicola* Deletion Mutants

Construction of deletion mutants was performed as described in Gonçalves et al. (2018). For *mtdh1*Δ and *mtdh2*Δ, the coding sequence (CDS) with ∼1 kb upstream and downstream were amplified from genomic DNA using the primer pairs listed in Supplementary Table S1. For the double mutant (*mtdh1Δmtdh2*Δ), the same strategy was used as the two genes are located next to each other in the genome separated by a small intergenic region (∼1 kb). The three disruption cassettes were subsequently used to transform *St. bombicola* PYCC 5882 as previously described in Gonçalves et al. (2018). Two different transformants from each gene disruption transformation were subsequently used for the phenotypic assays.

For the construction of *mtdh1*Δ*sor1*Δ, the *SOR1* gene was searched in the genome of *St. bombicola* PYCC 5882 using *Y. lipolytica* sorbitol dehydrogenase as query (XM_503864.1). The putative *SOR1* gene was deleted in the *mtdh1:HYG* background using a zeocin resistance cassette (consisting in the *ble* gene from *Staphylococcus aureus* under control of the *GPD* promoter from *St. bombicola* and the *CYC* terminator from *Saccharomyces cerevisiae*). Transformants were selected in YPD plates (pH = 6.8) supplemented with 650 μg/mL zeocin (Invivogen, San Diego, CA, United States). For the construction of the *hxk1*Δ*glk1*Δ mutant, *HXK1* and *GLK1* from *S. cerevisiae* (YCL040W and YFR053C) were used to search for the respective orthologs in *St. bombicola* PYCC 5882 genome. A similar strategy as aforementioned was employed, starting with the construction of the *glk1:ZEO* mutant followed by disruption of the *HXK1* in the g*lk1:ZEO* background using a hygromycin resistance cassette.

### Enzymatic Assays

For the preparation of cell-free extracts, the cells were collected by centrifugation (3,000 × *g* for 5 min), washed twice with cold 100 mM Tris-HCl buffer (pH = 7.5) and disrupted with glass beads in 500 μL of Lysis Buffer (0.1 M triethanolamine hydrochloride, 2 mM MgCl_2_, 1 mM DTT and 1 μM PMSF) with six cycles of 60 s vortex-ice. Cell debris were removed by centrifugation at 4°C, 16,000× *g* for 20 min and the cleared raw extracts were stored at −20°C. Protein was quantified using the QUBIT fluorimeter following the manufacturer’s guidelines. The mannitol dehydrogenase (Mtdh) assay was performed in both the reductive and the oxidative directions at 25°C. For the oxidative reaction, 500 μL of reaction mixture was used containing 50 mM Tris-HCl (pH = 8.5), 1 mM of NADH or NADPH and 25 μL of protein extract (200–500 ng/μL of total protein). The reaction was started by the addition of fructose to a final concentration of 200 mM and oxidation of NADH or NADPH was monitored by the decrease in absorbance at 340 nm. For the reductive reaction, 50 mM of Tris-HCl (pH = 10) buffer and 1 mM of NAD^+^ or NADP^+^ were used instead. The reaction was started by adding mannitol to a final concentration of 50 mM and reduction of NAD^+^ or NADP^+^ was monitored by the increase in absorbance at 340 nm.

For the hexokinase assay (used as a control for the quality of the extracts when testing for the lack of Mtdh activity in the *St. bombicola mtdh* deletion mutants), a reaction mixture was prepared containing 50 mM Tris buffer (pH = 7.5), 10 mM MgCl_2_, 1 mM ATP, 1 mM NADP^+^, 0.2 U of glucose-6-P dehydrogenase (Sigma Aldrich, St. Louis, MO, United States) and 1 U phosphoglucose isomerase (Sigma Aldrich, St. Louis, MO, United States). Enzymatic assays were performed at 25°C in a total volume of 500 μL and 25 μL of cell-free extract (200–500 ng/μL of total protein) was used. The reaction was started by the addition of fructose to a final concentration of 100 mM and reduction of NADP^+^ was monitored spectrophotometrically by the increase in absorbance at 340 nm for 2 min.

For the detection of NADP^+^-dependent glycerol dehydrogenase activity a reaction mixture containing 50 mM Tris-HCl (pH = 8.5) buffer, 1 mM NADP^+^ and 25 μL of cell-free extract was prepared. The reaction was started by the addition of 100 mM of glycerol, and NADPH formation was monitored spectrophotometrically for 2 min.

For the detection of glucose isomerase activity, a protocol based on Sayyed et al. (2010) was used. Cells of both the wild type *St. bombicola* and the *mtdh1*Δ*mtdh2*Δ mutant were cultivated for 24 or 76 h in YP supplemented with 20% (w/v) of glucose and cell-free extracts were obtained as described above. Glucose isomerase activity was measured in a 2.4 mL reaction mixture containing 20–50 μg of total protein, 0.5 mL 50 mM of KPi (pH = 7.2), 2 mM CaCl_2_, 20 mM glucose, 10 mM MgSO_4_.7H_2_O. Different temperatures (25, 30, and 60°C) and reaction times (1, 3, and 24 h) were tested. The eventual formation of fructose was assessed by HPLC.

For the detection of fructose-6-phosphate phosphatase activity, the *mtdh1*Δ*mtdh2*Δ mutant was grown in YP medium supplemented with 20% (w/v) glucose for 24 h at 25°C. Cell-free extracts were obtained as previously described. A solution containing 20–50 μg of total protein was incubated at 25°C in 100 mM of Tris Buffer (pH = 7.5) for 3 min. The reaction was started by the addition of fructose-6-phosphate to a final concentration of 50 mM or glucose-6-phosphate (used as a control) and left to proceed for 25 min. Two controls were also included, one in which no substrate was added and another one in which no extract was added. 300 μL samples were taken at different time points (0, 5, 10, and 20 min) and were immediately subjected to a procedure for the detection of inorganic phosphate based on Ames (1966). Briefly, 700 μL of a reagent mix (consisting in six parts of a solution of 0.42% (w/v) ammonium molybdate prepared in 1 N H_2_SO_4_ and one part of a solution of 10% (w/v) ascorbic acid prepared in milli-Q water) was added to each of the samples. The reaction was left to proceed for 1 h at 37°C. Absorbance at 820 nm was then measured.

### Growth Assays and Sugar Consumption

For growth assays, *St. bombicola* wild type and *MtDH* deletion mutants were pre-grown overnight in 100 mL flasks containing 20 mL of culture medium at the temperature of choice. This inoculum was diluted to OD_640_ = 0.2 in 30 mL of YP (1% (w/v) yeast extract and 2% (w/v) peptone) supplemented with the different carbon sources tested in 250 mL flasks. Growth was monitored for 96–150 h.

To assess sugar consumption and metabolite production, wild type, *mtdh1*Δ and *mtdh1*Δ*mtdh2*Δ were grown as described before in YP supplemented with 10% (w/v) glucose and 10% (w/v) fructose at 30°C. Samples of 1 mL were collected from culture supernatants, centrifuged at 16,000 x *g* for 1 min and analyzed by HPLC as described previously. Data was visualized using GraphPad Prism version 6 for Windows, GraphPad Software, San Diego, CA, United States, www.graphpad.com.

### Quantification of Extracellular and Intracellular Metabolites

*Starmerella bombicola* wild type was cultivated for 72 h in 30 mL of YP medium supplemented with 10% (w/v) fructose and 10% (w/v) glucose (20FG medium) at 20, 25, and 30°C at 180 r.p.m. After this period, cells were harvest by centrifugation (4°C, 10 min, 8,000 × *g*) and 1 mL of supernatant was collected to quantify extracellular metabolites by HPLC as described previously.

Cell-free extracts for intracellular metabolite analysis were obtained as described for the enzymatic assays. The samples were filtered using a nylon filter with a pore diameter of 0.2 μm and subsequently eluted with 0.6 M NaOH at 0.4 mL/min at 25°C in a CarboPac MA1 column and the peaks were identified with a pulsed amperometric detector.

To determine the total dry weight 2 mL of culture broth were centrifuged for 5 min at 9,000 × *g*. Pellets were washed twice and resuspended in distilled water. The suspension was filtered onto a nitrocellulose filter with a pore diameter of 0.45 μm and was dried at 80°C until a constant weight was attained.

To estimate the intracellular concentration of mannitol per cell (mM), the total amount of mannitol in the cell-free extracts was determined and divided by the number of cells used to produce the extract. We estimate that the volume of one cell of *St. bombicola* would be ∼4 × 10^–10^ mL [half the volume of *S. cerevisiae*, Zakhartsev and Reuss (2018)]. Statistical significance was tested using a one-way ANOVA using the Bonferroni’s correction for multiple testing, implemented in GraphPad Prism v6.

### ^13^C-NMR Analysis of ^13^C-Labeled Glucose Metabolism by Resting Cells

Cells were grown in YP medium supplemented with 20% (w/v) glucose (YP20G) for 24 h at 30°C. Cells were harvested, washed twice with 50 mM KPi buffer (pH = 7) and kept on ice. The cells were resuspended in 5 mL of KPi buffer (50 mM, pH = 7), to a final OD_640_∼50. The cell suspension was incubated at 30°C and approximately 50 mM of [1-^13^C]glucose or [2-^13^C]glucose was provided at time point zero; aliquots of 750 μL were withdrawn at different time points (0, 15, 30, 45, 60, and 120 min). The collected samples were centrifuged, and the supernatant solutions stored at −20°C until further analysis. NMR samples were prepared by adding 75 μL of deuterated water containing trimethylsilylpropanoic acid (TSP, final concentration of 0.44 mM) to 475 μL of each supernatant solution. All spectra were acquired at 30°C in a Bruker Avance II^+^ 500 spectrometer, equipped with a 5 mm TCI H/C/N Prodigy Cryoprobe. For each sample the following spectra were acquired: one-dimensional ^1^H-NMR (noesygppr1d, 64 free induction decays, with 64 k complex points, spectral window of 20 ppm, 5 s relaxation delay and 10 ms mixing time); ^13^C-NMR with inverse gated proton decoupling (zgig, 1k free induction decays, with 64 k complex points, spectral window of 248.5 ppm and 60 s relaxation delay). Whenever needed for spectral assignment, extra spectra were acquired: quantitative ^1^H-NMR spectra (zgprde, 64 free induction decays, with 64 k complex points, spectral window of 20 ppm, 4 s relaxation delay and 60 s extra relaxation delay) and two-dimensional ^13^C-^1^H HSQC spectra (hsqcetgpsisp2 pulse sequence, 256 points in F1 and 2048 points in F2; 32 free induction decays; relaxation delay of 1.5 s; sweep width of 25153.695 Hz in F1 and 8012.820 Hz in F2). Spectra acquisition and processing was performed using TopSpin 3.2. The concentration of β-[2-^13^C]glucose and [2-^13^C]ethanol was determined in the fully relaxed ^1^H-NMR spectra using the TSP signal as an internal concentration standard; this information was used to determine ^13^C concentrations from ^13^C-NMR spectra. Assignment of spectra was performed resorting to the HMDB^1^ and BMRB^2^ databases.

### Analysis of Metabolic Fluxes

The essential features of the model required for flux analysis were determined by the metabolites which were observed (shown in bold in Figure 3B) and the standard routes into the TCA cycle. The appearance of significant amounts of fructose with reversed label (e.g., [6-^13^C]fructose from [1-^13^C]glucose) required the inclusion of a reverse flux from the triose pool to fructose. Numerical values were obtained by assuming that the fructose concentration reached an approximate steady state after 30 min, i.e., that the fluxes into and out of the fructose pool are then equal. The ratio of original and reversed label is then given by (2 × f_*IN*_ + f_*R*_)/f_*R*_, where f_*R*_ is the back-flux and f_*IN*_ is the inward flux of labeled glucose, obtained from its approximately linear consumption.

The flux through the pentose phosphate pathway (PPP) was obtained from the concentrations of [1-^13^C]fructose and [1,3-^13^C]fructose observed in experiments starting with [2-^13^C]glucose. In the first cycle of PPP, three molecules of [2-^13^C]glucose will produce one of [1-^13^C]fructose-6-P and one of [1,3-^13^C]fructose-6-P. The mole fractions of fructose with carbon 1, 2 or 5 labeled (m_1_, m_2_ and m_5_) are approximately constant, and reflects its origin either directly from [2-^13^C]glucose (with back-flux) or via PPP. The flux into PPP is then 1.5 m_1_f_*IN*_/(1.5 m_1_ + m_2_ + m_5_), allowing for the production of two fructose molecules from three of glucose in PPP. Consumption of [1,3-^13^C]fructose in a second PPP cycle produced a small amount of [2,3-^13^C]fructose which was not used in the estimation of fluxes. A small but significant amount of [3-^13^C]fructose was also generated but its origin remains obscure.

## Results

### Identification of Putative Mannitol Biosynthetic Genes in W/S-Clade Species

**FIGURE 1:**
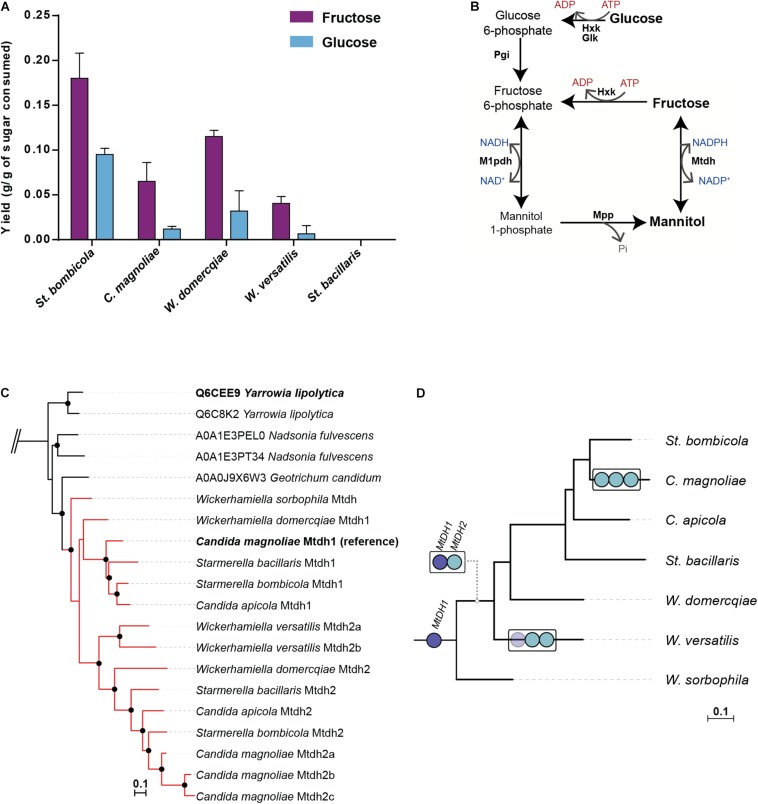
Mannitol production and phylogenetic analyses of mannitol-related genes in W/S-clade species. **(A)** Mannitol yields determined after 72–150 h of growth on YP medium supplemented with 10% (w/v) glucose or 10% (w/v) fructose at 30°C in several W/S-clade species (*Starmerella bombicola*, *Candida magnoliae*, *Starmerella bacillaris*, *Wickerhamiella domercqiae* and *Wickerhamiella versatilis*). Assays were performed in duplicate and the error bars represent the standard deviation. **(B)** Mannitol biosynthetic pathways in Fungi as described by Hult et al. (1980). Glk – Glucokinase, Hxk – Hexokinase, Pgi – Phosphoglucose isomerase, Mtdh – mannitol dehydrogenase, Mpp – mannitol-1-phosphate phosphatase, M1pdh – mannitol-1-phosphate dehydrogenase. **(C)** Pruned Maximum Likelihood (ML) phylogeny of Mtdh-like proteins using the top 750 hits (UniprotKB database) to *St. bombicola* Mtdh1. Branches with support higher than 95% are indicated by black dots. W/S-clade proteins are shown in red. Complete phylogeny can be accessed in Figshare 10.6084/m9.figshare.9959693. **(D)** Schematic representation of the phylogenetic relationships between W/S-clade species based on Gonçalves et al. (2018). *MtDH* genes are represented by blue circles (dark blue- *MtDH1*, light blue- *MtDH2*). The duplication events are represented by white boxes in the respective branches in which they were assumed to have occurred. In *W. versatilis*, *MtDH1* seems to have been lost (faded dark blue circle).

Mannitol production in appreciable amounts, an unusual feature in yeasts, was previously detected in at least one W/S-clade species, *C. magnoliae* (Baek et al., 2003; Lee et al., 2003). To find out whether mannitol production is a widespread trait in the W/S clade, we quantified mannitol in the culture supernatants of representatives of five W/S-clade species, namely *C. magnoliae*, *St. bacillaris*, *St. bombicola*, *W. domercqiae* and *W. versatilis*, cultivated in rich medium containing either 10% glucose or 10% fructose as carbon and energy source. We found that all species but one produced variable amounts of mannitol in addition to ethanol and glycerol, and that more mannitol was produced from fructose than from glucose (Figure 1A). In *St. bacillaris* neither of the two strains tested (PYCC 3044 and PYCC 6282) produced mannitol; in this species glycerol, erythritol and ethanol were produced instead.

In fungi, mannitol can be produced from fructose through two distinct pathways. The first, usually found in filamentous fungi (Solomon et al., 2007), involves the NADH dependent conversion of fructose-6-phosphate (fructose-6-P) to mannitol-1-phosphate (mannitol-1P), followed by dephosphorylation to yield mannitol. The second, previously shown to operate in the oleaginous yeast *Y. lipolytica* (Napora et al., 2013), involves the direct conversion of fructose to mannitol through an NADPH dependent mannitol dehydrogenase (Figure 1B). The two pathways may coexist, in which case they form a cycle that may interconvert NAD(H) and NADP(H) (Hult et al., 1980). The fact that W/S-clade species produce mannitol when glucose is the only carbon and energy source, suggests that the pathway involving the conversion of fructose-6-P to mannitol-1P is operating in these yeasts, since the direct conversion pathway is specific for fructose. This specificity was demonstrated for the purified mannitol dehydrogenase from W/S-clade species *C. magnoliae* (Lee et al., 2003).

To obtain a first insight into the pathway(s) involved in mannitol production in the W/S clade, the genomes of six W/S-clade species were surveyed for the presence of genes likely to encode components of the mannitol biosynthetic pathways. Hence, tBLASTx searches were performed using available fungal protein sequences for the various components of the mannitol biosynthetic pathways as queries. The results (Supplementary Table S2) showed that genes encoding mannitol-1P dehydrogenase and mannitol-1P phosphatase seem to be absent in all genomes examined, leading to the unanticipated conclusion that in the W/S clade biosynthesis of mannitol from glucose most probably does not proceed through the pathway involving these two enzymes. On the contrary, homologs of the *Y. lipolytica* mannitol dehydrogenase (*YlSDR*) gene responsible for the direct conversion of fructose to mannitol (Napora et al., 2013; Dulermo et al., 2015) were readily found in all genomes examined. Among the genes identified was the previously characterized *MtDH1* from *C. magnoliae* (Lee et al., 2003). In *St. bombicola*, we also found next to *MtDH1* gene (Supplementary Figure S1), a second, very similar gene (encoding a protein with ∼63% similarity to Mtdh1) that we named *MtDH2*.

To shed light on the evolutionary origin of the Mtdh-like proteins identified in W/S-clade genomes, a ML phylogeny was constructed including all the Mtdh proteins identified in W/S-clade genomes as well as the 750 best BLASTp hits in UniprotKB (reference proteomes database) obtained using *St. bombicola* Mtdh1 as a bait (Figure 1C). This phylogeny suggests that Mtdh1 and Mtdh2 are derived from a duplication event that took place in a common ancestor of the W/S clade (Figure 1D). Each examined species possesses a single gene of each type, with the exception of *C. magnoliae*, which possesses three *MtDH2*-like genes and *W. versatilis* that lacks the *MtDH1* gene. *Wickerhamiella sorbophila* (formerly *Candida infanticola)* does not exhibit the duplication (Figures 1C,D).

### Direct Conversion of Fructose to Mannitol in W/S-Clade Yeasts

Comparative genomic analyses showed in the previous section that W/S-clade genomes encoded two (or more) putative paralogous mannitol dehydrogenases, which potentially catalyze the interconversion between fructose and mannitol. To consubstantiate this finding and demonstrate that mannitol is produced directly from fructose in W/S-clade species, we assayed Mtdh activity in cell-free extracts of the species under study. In line with the absence of mannitol in culture supernatants, no Mtdh activity was detected in *St. bacillaris* irrespective of the cofactor (NADH or NADPH) used, although the *MtDH1* and *MtDH2* genes are also present in this species (Figure 1C and Supplementary Figure S1). For all other W/S-clade species, NADPH-dependent Mtdh activity was readily detected (Supplementary Figure S2), while no activity could be measured when NADH was supplied as a cofactor. *St. bacillaris* does not produce mannitol but rather glycerol and erythritol. Interestingly, in *St. bacillaris* glycerol synthesis involves NADP^+^ recycling since it requires NADPH as cofactor (Supplementary Figure S2B) while in the other W/S-clade species this cofactor was apparently not used.

### Genetic Dissection of Mannitol Production in *St. bombicola*

**FIGURE 2:**
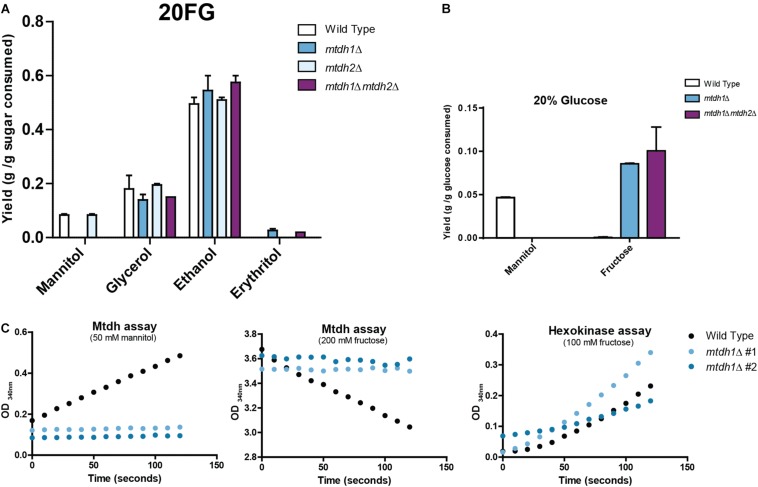
Metabolite production and mannitol dehydrogenase (Mtdh) activities in *St. bombicola* wild type and deletion mutants. **(A)** Metabolite production in *St. bombicola* wild type and deletion mutants (*mtdh1*Δ, *mtdh2*Δ, *mtdh1*Δ*mtdh2*Δ) cultivated in YP supplemented with 10% (w/v) glucose and 10% (w/v) fructose (20FG) at 30°C for 76 h. **(B)** Mannitol and fructose production in *St. bombicola* wild type, *mtdh1*Δ, *mtdh1*Δ*mtdh2*Δ cultivated in YP medium supplemented with 20% (w/v) glucose at 30°C for 76 h. **(C)** Mtdh activities in cell-free extracts of wild type *St. bombicola* (black dotted line) and *mtdh1*Δ (blue lines represent two biological replicates). The hexokinase assay served as a control for the quality of the extracts.

In order to find out which of the two *MtDH* genes, if any, was responsible for mannitol production in W/S-clade yeasts, single and double *MtDH* deletion mutants were constructed in the genetically amenable species *St. bombicola*, which encodes one Mtdh1 and one Mtdh2. As shown in Figure 2, in the *mtdh1*Δ and *mtdh1*Δ*mtdh2*Δ mutants no mannitol was detected in culture supernatants (Figure 2A) and no mannitol dehydrogenase activity could be measured in cell free extracts of *mtdh1*Δ (Figure 2C), suggesting that Mtdh1 is the main enzyme responsible for mannitol synthesis in this yeast. This agrees with the fact that mannitol biosynthesis seems to be unaffected in the *mtdh2*Δ mutant (Figure 2A).

Notably, mannitol production from glucose is also hampered in the mutants lacking *MtDH1* (Figure 2B). This result can only be explained by the existence of a yet unknown pathway mediating conversion of glucose to mannitol. Our genetic evidence suggests that such a pathway must include the reaction catalyzed by Mtdh1. Corroborating this, we found considerable amounts of fructose (∼10 g/L; Figure 2B) in the culture supernatants of *mtdh1*Δ and *mtdh1*Δ*mtdh2*Δ mutants cultivated on glucose. Therefore, we conclude that fructose is an intermediate in a novel pathway responsible for the biosynthesis of mannitol from glucose in *St. bombicola* and that fructose accumulates and is excreted when the fructose —> mannitol conversion is genetically prevented.

### Conversion of Glucose to Fructose in W/S-Clade Yeasts

To examine whether the observed conversion of glucose to fructose was likely to be occurring as well in wild type *St. bombicola*, we examined the culture supernatants of the wild type strain for the presence of fructose. Although in much lower amounts than in the *mtdh1*Δ mutant (∼0.24 g/L in the wild type vs. ∼10 g/L in the mutant), fructose could also be detected in the supernatants of the wild type strain cultivated on 200 g/L glucose. The same was observed for wild type *W. versatilis* (1 g/L fructose detected by HPLC after ∼200 h of growth at 30°C), another W/S-clade species that produces mannitol (Figure 1A), showing that the glucose to fructose conversion was not an artifact of the *St. bombicola* mutant. Instead, this reaction seems to be part of a new pathway likely shared by W/S-clade yeasts that produce mannitol from glucose.

Conversion of glucose to fructose has not been described in yeasts so far but many bacteria and a few fungal species conduct this direct conversion using a glucose isomerase, an enzyme of exceptional biotechnological interest (Bhosale et al., 1996). Examples in fungi are found in species belonging to the genera *Piromyces* (Lee et al., 2017), *Aspergillus* (Sayyed et al., 2010) and *Penicillium* (Kathiresan and Manivannan, 2006). We searched for the presence of a glucose isomerase in the *St. bombicola* genome and the genomes of other W/S-clade species using fungal and bacterial glucose isomerases as queries in tBLASTx searches (AJ249909.1 and FJ858195.1, respectively), but no candidate genes could be found (*e*-value >1e^–3^, Supplementary Figure S3). Importantly, we also failed to detect glucose to fructose conversion in cell free extracts.

Since no evidence could be found for the presence of a gene encoding a glucose isomerase, we searched for other known biochemical routes that could potentially be used by *St. bombicola* to produce fructose from glucose. A two-step conversion can be envisioned involving an aldose reductase to convert glucose to sorbitol and then a sorbitol dehydrogenase to convert sorbitol to fructose (Supplementary Figure S3A), a pathway that has been previously described (Muntz and Carroll, 1960). A candidate gene for a sorbitol dehydrogenase could be readily retrieved from the genome of *St. bombicola* by tBLASTx using a sorbitol dehydrogenase sequence from *Y. lipolytica* (YALI0E12463, XM_503864.1) while the results for the aldose reductase (XM_502540.1) were not clear (Supplementary Figure S3B). Nevertheless, disruption of the sorbitol dehydrogenase (*SOR1*) gene in *St. bombicola* was performed and we observed that this mutant was unable to grow on sorbitol as carbon source confirming the presumed function of the *St. bombicola* Sor1 protein. However, deletion of *SOR1* in the *mtdh1*Δ mutant background (*mtdh1*Δ*sor1*Δ) did not affect glucose conversion to fructose, since similar levels of fructose were measured in the supernatants of the *mtdh1*Δ and *mtdh1*Δ*sor1*Δ mutants (∼10 g/L and ∼8 g/L, respectively) leading to the conclusion that this pathway is not involved in the conversion. Finally, we considered the possibility that fructose might be generated by dephosphorylation of the glycolytic intermediate fructose-6-phosphate (fructose-6P). Very few references are found in the literature to fructose-6P phosphatases in fungi but the activity has been studied in connection with mannitol metabolism in mushrooms (Kulkarni, 1990). Since genes that can be used as baits in tBLASTx searches are unavailable, we decided to use ^13^C-labeled glucose and NMR analysis of end-products to obtain definite evidence for the occurrence of this novel metabolic pathway.

### A Fructose-6-Phosphate Phosphatase Is the Key to Mannitol Production From Glucose

**FIGURE 3:**
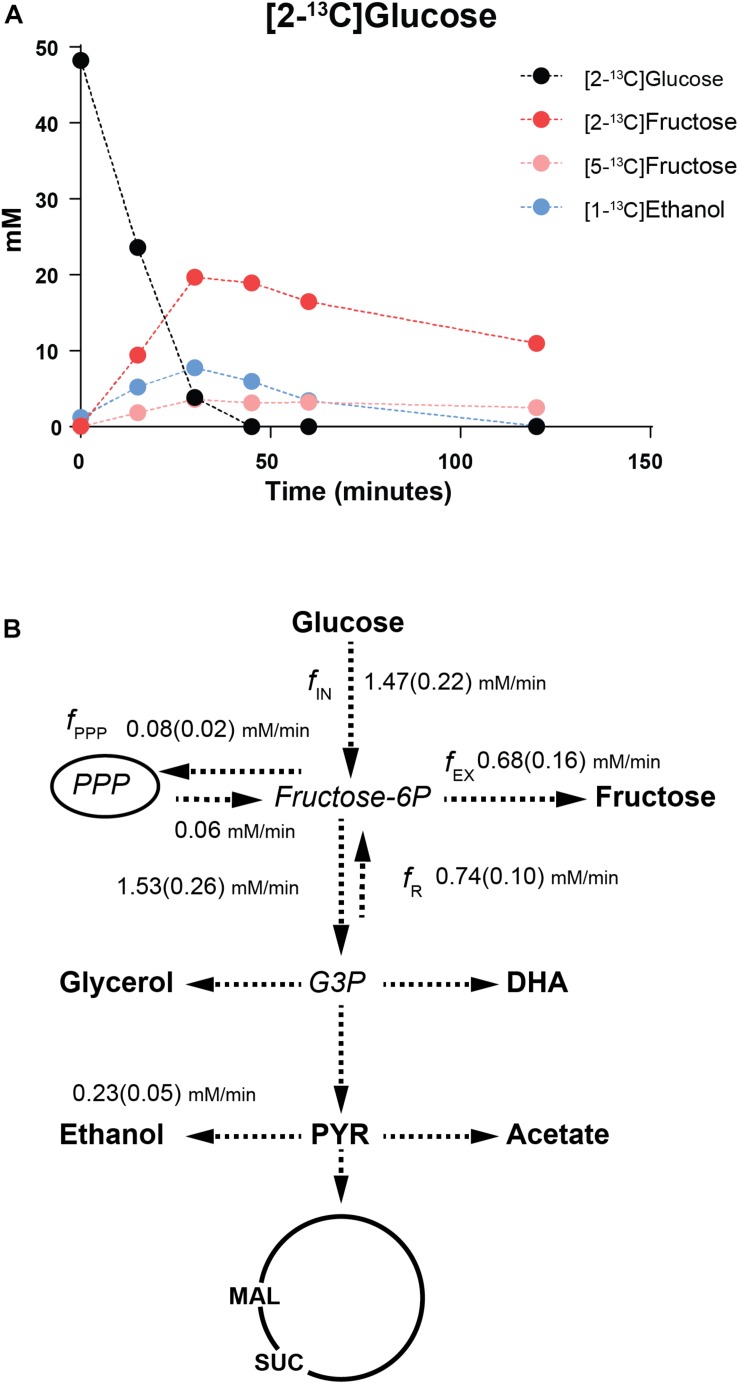
^13^C-NMR analyses of [2-^13^C]glucose metabolism in *St. bombicola mtdh1*Δ*mtdh2*Δ mutant. **(A)** Time course of ^13^C-labeled glucose consumption and product formation by resting cell suspensions of the *St. bombicola mtdh1*Δ*mtdh2*Δ mutant. End products were quantified by ^13^C-NMR in supernatants after a pulse (at time zero) of [2-^13^C]glucose to a final concentration of approximately 50 mM. A comprehensive list of labeled end-product concentrations for this and similar assays is shown in Supplementary Table S3. **(B)** Metabolic fluxes deduced from experiments with ^13^C-labeled glucose. Observed metabolites are shown in bold (DHA: dihydroxyacetone; PYR: pyruvate; MAL: malate; SUC: succinate) and inferred intermediates in italic (G3P: glyceraldehyde-3-phosphate). PPP represents the reactions of the pentose phosphate pathway. Each conversion, shown by dashed lines, may involve multiple steps. Standard deviations are shown in brackets.

To trace the metabolic fate of glucose in the *mtdh1*Δ*mtdh2*Δ mutant, where considerable accumulation of fructose in the growth medium was observed, we first established that resting cells of this mutant, pre-grown on glucose and resuspended in phosphate buffer, produced ∼3 g/L of fructose when given a glucose pulse (∼50 mM). We subsequently performed similar experiments but using pulses of ^13^C-glucose labeled on C_2_ ([2-^13^C]glucose) or labeled on C_1_ ([1-^13^C]glucose) to allow for tracing the metabolic fate of glucose by NMR. The label from [2-^13^C]glucose was retrieved mainly in fructose and ethanol (Figure 3A and Supplementary Table S3); minor amounts of [2-^13^C]pyruvate, [1-^13^C]acetate, [2-^13^C]glycerol and [2-^13^C]dihydroxyacetone were also detected (Supplementary Table S3). If a direct conversion of glucose into fructose would occur, [1-^13^C]glucose and [2-^13^C]glucose would yield exclusively [1-^13^C]fructose and [2-^13^C]fructose, respectively. However, the fructose pool contained a substantial fraction (∼10%) of [6-^13^C]fructose derived from [1-^13^C]glucose and [5-^13^C]fructose from [2-^13^C]glucose (Figure 3A and Supplementary Table S3). This label diversion implies the occurrence of a reversed flux from the triose phosphate pool toward fructose-1,6-bisphosphate through aldolase (Supplementary Figure S4) and has been amply illustrated in early *in vivo* ^13^C-NMR studies of central metabolism in *Lactococcus lactis* (Neves et al., 1999). This reversed flux is especially apparent when there is a metabolic constraint such as that created by a deficiency in activities that are key for the fulfillment of the redox balance (Neves et al., 2000). In the case of *St. bombicola* the observed label diversion unequivocally shows that there is fructose-6P phosphatase activity in addition to the gluconeogenic enzyme, fructose-bisphosphatase; unfortunately, we were unable to detect such activity in crude cell-free extracts of the *mtdh1*Δ*mtdh2*Δ using a standard methodology for detection of inorganic phosphate (Ames, 1966).

While these results show that a phosphatase is implicated in the conversion of glucose to fructose in *St. bombicola*, they do not exclude the involvement of an isomerase (see Supplementary Figure S4). To investigate this, we constructed a *St. bombicola* mutant in which the hexokinase (*HXK1*) and glucokinase (*GLK1)* genes were disrupted, completely precluding hexose phosphorylation. We envisaged that in this mutant, glucose to fructose conversion could still take place only if an isomerase was involved, but not if a fructose-6P phosphatase was required (Supplementary Figure S5A). When the *hxk1*Δ*glk1*Δ mutant was grown in 10 g/L glycerol and 10 g/L fructose, mannitol was normally produced yielding ∼10 g/L (Supplementary Figure S5B). On the other hand, when the mutant was grown in a mixture of glycerol and glucose no mannitol or fructose production and no glucose consumption were observed (Supplementary Figure S5B), indicating that the inability to produce fructose-6P impedes mannitol formation from glucose and that the direct conversion of glucose to fructose, if present, is negligible and undetectable using this methodology. Altogether, our results led us to postulate that in *St. bombicola* glucose is converted into fructose via the intermediate metabolites glucose-6P and fructose-6P and the action of a yet unknown fructose-6P phosphatase.

On the basis of the ^13^C-labeling patterns observed on the fructose pool the relevant carbon fluxes around the fructose-6P node were estimated (Figure 3B; for details on flux analysis see section “Materials and Methods”). Glucose was consumed at a rate of 1.47 ± 0.22 mM/min while fructose was produced at a rate of 0.68 ± 0.16 mM/min. The flux from fructose-6P toward the Pentose Phosphate Pathway (f_*PPP*_) was small, only 0.08 mM/min. On the other hand, the reversed flux from the triose phosphate pool to fructose-6P was about half (48%) of the forward glycolytic flux. It is worth noting that there is a substantial flux from fructose-6P to fructose, in fact amounting to 86% of the net flux from fructose-6P to glycolysis.

### Mannitol Production Contributes to Fructophily

**FIGURE 4:**
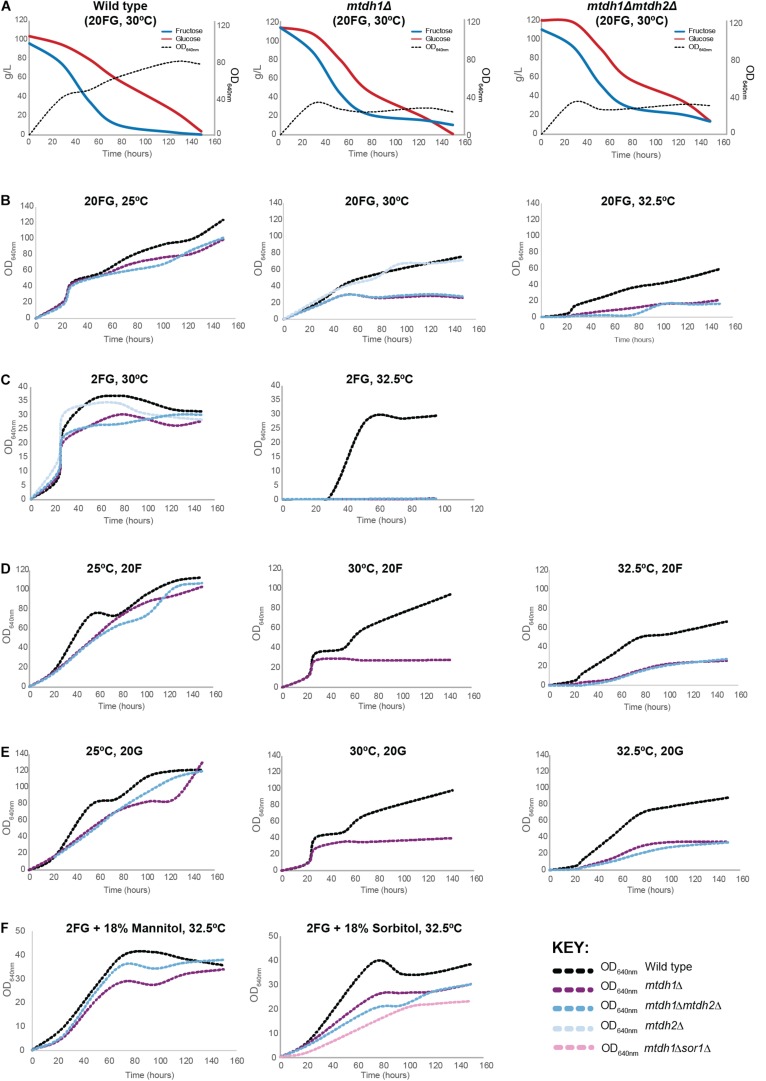
Effect of *MtDH* deletion on fructophily and on biomass yield at different growth temperatures. **(A)** Sugar consumption profiles of *St. bombicola* wild type, *mtdh1*Δ and *mtdh1*Δ*mtdh2*Δ mutants. Strains were cultivated in YP supplemented with 10% (w/v) glucose and 10% (w/v) fructose (20FG) for 150 h at 30°C. Experiments were performed with two biological replicates. **(B)** Growth of wild type, *mtdh1*Δ, *mtdh2*Δ and *mtdh1*Δ*mtdh2*Δ in YP supplemented with 10% (w/v) glucose and 10% (w/v) fructose (20FG) for 150 h at different temperatures: 25, 30, and 32.5°C. **(C)** Growth of *St. bombicola* wild type, *mtdh1*Δ and *mtdh1*Δ*mtdh2*Δ in YP supplemented with 1% (w/v) glucose and 1% (w/v) fructose (2FG) at 30 and 32.5°C. **(D,E)** Growth of *St. bombicola* wild type, *mtdh1*Δ and *mtdh1*Δ*mtdh2*Δ in YP supplemented with 20% (w/v) fructose (20F) **(D)** or 20% (w/v) glucose (20G) **(E)** for 150 h at 25, 30, and 32.5°C. **(F)** Growth of *St. bombicola* wild type, *mtdh1*Δ, *mtdh1*Δ*mtdh2*Δ in (2FG) supplemented with 18% (w/v) mannitol (left) or 18% (w/v) sorbitol (right) at 32.5°C. *St. bombicola mtdh1*Δ*sor1*Δ was only grown in 2FG supplemented with 18% (w/v) sorbitol. Raw data can be found in Supplementary Data S1.

As shown in the previous sections, mannitol production seems to be a key reaction in the metabolism of W/S-clade yeasts, which is best illustrated by the diversion of fructose-6P to mannitol production during growth on glucose. Mannitol production from glucose demands ATP expenditure but this is not the case when fructose is the substrate (Figure 1B). This is in line with the higher mannitol yield obtained from fructose when compared to the mannitol yield when glucose is used as the sole carbon source (Figure 1A) and could explain why fructose is preferred over glucose in these yeasts. Hence, we set out to investigate whether deletion of the *MtDH1* gene affected fructophily, by cultivating the mutant under the conditions where fructophily is usually observed (YP medium supplemented with 10% glucose + 10% fructose; henceforth referred to as 20FG). The results, shown in Figure 4A revealed an effect of the *MtDH1* gene deletion on the consumption rates of both fructose and glucose, decreasing the first and increasing the latter, which results in attenuation of the preference for fructose. In the culture supernatants of the two mutants that fail to produce mannitol (*mtdh1Δ* and *mtdh1*Δ *mtdh2*Δ), small amounts of erythritol were detected, while ethanol and glycerol yields on sugar remained virtually unaltered (Figure 2A). Since erythritol production regenerates NADP^+^, this result suggests that abolition of mannitol synthesis causes a redox imbalance at least partly compensated by an increase in erythritol production.

### A Possible Role for Mannitol in Thermal Protection

Loss of the ability to synthesize mannitol was accompanied by a drop in the biomass yield on sugar, when compared with the wild type (Figure 4A). We first hypothesized that this decrease in biomass yield in the mutants incapable of producing mannitol could be due to a role of mannitol as a compatible solute, required to cope with osmotic stress in the high sugar concentration medium. In line with this possibility, we observed that the *mtdh1*Δ and *mtdh1*Δ *mtdh2*Δ mutants grew as well as the wt in medium containing 2% sugar (1% glucose + 1% fructose; henceforth referred to as 2FG), attaining similar cell densities (Figure 4C). However, when we grew the mutants in low sugar concentrations (2FG) but at 32.5°C instead of the previous 30°C, the *mtdh1*Δ and *mtdh1*Δ*mtdh2*Δ mutants did not grow (Figure 4C), suggesting that mannitol might rather play a role in adaptation to thermal stress.

To assess the possibility that mannitol might function as a thermal protector in *St. bombicola*, we revisited the performance of the single and double *mtdh* mutants in high sugar medium (20FG), at three different temperatures. The results, shown in Figure 4B demonstrate that also in 20FG medium, the growth defect is more pronounced at 32.5°C than at 30°C, although in this medium the mutants are still capable of growing at 32.5°C. Since at 25°C, only a mild effect of the inability to synthesize mannitol on growth was observed (Figure 4B) irrespective of the osmolarity of the growth medium it seems unlikely that mannitol plays a major role in osmoprotection. The effects produced by the increase in temperature were not specific for one of the sugars in particular (Figures 4D,E).

Next, we examined whether mannitol production increased with temperature in the wild type, which we anticipated should occur if it has a thermo-protective role. Relative internal (in cell-free extracts) and external mannitol concentrations were measured at different temperatures. An increase in extracellular mannitol concentration was readily observed with increasing temperatures (Supplementary Figure S6A), which does not happen for other fermentation products like ethanol and glycerol (Supplementary Figure S6B). We estimated intracellular mannitol concentrations per cell to vary between 5 and 40 mM, which might be sufficient to fulfill a role in thermal protection, but we did not see a clear increase in intracellular concentrations (Supplementary Figures S6C,D). Nevertheless, this represents a small fraction (approximately 1–5%) of the total amount of mannitol produced.

*Starmerella bombicola* does not grow well on mannitol, requiring up to 1 week of lag phase, but since it does eventually grow, this means that it possesses a transport system that enables it to take up mannitol. We reasoned that if mannitol itself, and not (only) the mannitol producing reaction was important for growth of the *mtdh1*Δ mutants at high temperatures, we might be able to ameliorate the growth defect by adding mannitol to the culture medium. In fact, when we added 18% (w/v) mannitol to the 2FG medium in which the mutants fail to grow at 32.5°C, the *MtDH1* mutants were able to achieve near wild type cell densities, albeit somewhat slower (Figure 4F). To find out whether this might also hold true for other polyols, we performed the same experiment using sorbitol, with similar results (Figure 4F). Moreover, when we used a mutant rendered unable to use sorbitol as carbon and energy source because of the deletion of a gene encoding an essential enzyme of sorbitol metabolism (*SOR1*) and lacking also *MtDH1*, growth was still observed (Figure 4F), showing that growth rescue is not dependent on sorbitol metabolization.

## Discussion

We showed recently that fructophily arose in yeasts in the W/S clade, a lineage that comprises yeast species sharing a recent common ancestor that lost the ability to conduct alcoholic fermentation. This central metabolic pathway was later reacquired by incorporation of an alcohol dehydrogenase of bacterial origin (Gonçalves et al., 2018). We also showed previously that the molecular basis for fructophily was established in a W/S-clade ancestor through the horizontal acquisition of a high capacity specific fructose transporter, Ffz1, from filamentous fungi (Gonçalves et al., 2016). Hence, two events with impact on metabolism, both unique in the evolutionary history of yeasts, took place in the same lineage and as far as can be currently judged, at not too distant a moment in evolution from each other. One pressing question arising from this observation is whether the two events might be somehow related. In order to explore this possibility, we sought to gather additional information on the fate of fructose in fructophilic yeasts, once it is taken up by the Ffz1 transporter. Here we showed that a significant amount of fructose is converted to mannitol in most W/S-clade yeasts tested.

### Role of the Mannitol Biosynthetic Pathway in the W/S-Clade

Contrary to what was suggested previously for the yeast *Y. lipolytica*, our evidence does not support a role for mannitol as transient carbon storage (Dulermo et al., 2015). The fact that most of the mannitol produced is excreted to the growth medium and not reutilized, and that the mannitol biosynthesis reaction is associated with cofactor recycling, rather suggests that mannitol is mainly a fermentation product and that its production has a role in redox balance. Consistent with this, we found that the pathway for mannitol production consists in a direct conversion of fructose to mannitol with NADP^+^ regeneration, mediated by an NADPH-dependent mannitol dehydrogenase and that all W/S-clade genomes examined lacked genes encoding the enzymes involved in the alternative pathway that converts fructose-6P to mannitol-1P. In *St. bombicola*, we obtained genetic evidence for the involvement of a mannitol dehydrogenase only, since elimination of *MtDH1* obliterates detectable mannitol production, notably also when glucose is the carbon source. Most importantly, we demonstrated that in spite of the absence of the mannitol-1P dehydrogenase pathway, glucose is also efficiently converted to mannitol through a novel pathway involving a fructose-6P phosphatase but not a glucose isomerase.

The role of a second gene, presumably paralogous to *MtDH1* could not be established with certainty. This gene, named *MtDH2* is phylogenetically closely related to *MtDH1* but its deletion has no measurable impact on the amount of mannitol produced. However, it seems likely that *MtDH2* has a role in mannitol metabolism in at least some W/S-clade species because *W. versatilis* lacks the *MtDH1* type of gene, possessing only several copies of the *MtDH2* type, but still produces mannitol. On the other hand, *St. bacillaris* has intact *MtDH1* and *MtDH2* genes but did not produce mannitol under the conditions tested, rather directing an important part of the carbon flux to glycerol. Therefore, either mannitol production occurs in this species under conditions distinct from those presently tested or these genes acquired a different function in *St. bacillaris*.

The fact that the production of the main polyol (either mannitol or glycerol) seems to be associated in W/S-clade species with NADP^+^ recycling suggests that their production plays a role in dealing with excess NADPH, which may result from the Pentose Phosphate Pathway (PPP) carrying a higher relative flux than for example in *S. cerevisiae*, where it seems to be finely tuned to the needs of NADPH for biosynthesis (Frick and Wittmann, 2005). In the oleaginous yeast *Y. lipolytica*, which is more closely related to the W/S clade, this pathway was shown to be overactive, providing also the NADPH required for lipid production (Wasylenko et al., 2015). The fact that erythritol was present in measurable amounts in the culture supernatant of the *mtdh* mutants but not of the wt, suggests a compensation as is normally observed in glycerol synthesis when ethanol production is impaired in yeasts (Drewke et al., 1990; Gonçalves et al., 2018).

Characterization of *St. bombicola* mutants unable to produce mannitol also suggested a role for mannitol as thermo protector, which we showed could be taken over by a similar compound as sorbitol. The growth rescue by external polyols is only partial, probably reflecting that redox balance is not optimal under these conditions. The effect of temperature on the growth defect phenotype of the *mtdh* mutants as well as the increase in mannitol production at higher temperatures consubstantiate the role of mannitol as thermo protector. Similar substances, like trehalose have been suggested to fulfill a thermo-protective role in *S. cerevisiae* by stabilizing the membrane when present on both sides (Magalhães et al., 2018). The fact that the *mtdh1*Δ mutants are capable of some growth at 32.5°C in 20FG medium but not in 2FG medium could suggest that the sugars, which are structurally similar to sugar alcohols, might contribute to ameliorate the thermo sensitive phenotype when present at higher concentrations (Corry, 1976; Henle et al., 1985).

### A New Pathway for the Conversion of Glucose Into Fructose Ensures Mannitol Biosynthesis in the Absence of Fructose

Taken together our findings point to mannitol playing an important role in central carbon metabolism in *St. bombicola* and also a possible role in stress protection (Wyatt et al., 2013), suggesting that fructose is preferred by this and other W/S-clade yeasts because it can be converted directly to mannitol. Our most striking finding that greatly emphasizes the importance of mannitol production for this yeast is the fact that it features a new pathway to convert glucose to fructose capable of carrying a considerable flux, therefore being able to operate the mannitol dehydrogenase reaction efficiently also when glucose is the only sugar available. In all documented cases so far in fungi, mannitol production from glucose relied on the mannitol-1P pathway (Solomon et al., 2007). As far as we could investigate taking advantage of the available W/S-clade genomes, the new pathway here reported is probably widespread in the W/S clade, a lineage that comprises presently close to 10% of all known yeast species, because all species examined are capable of producing mannitol from glucose and the only pathway for mannitol production seems to involve the fructose-specific Mtdh.

### A Possible Link Between Mannitol Metabolism and Evolution of Alcoholic Fermentation

**FIGURE 5:**
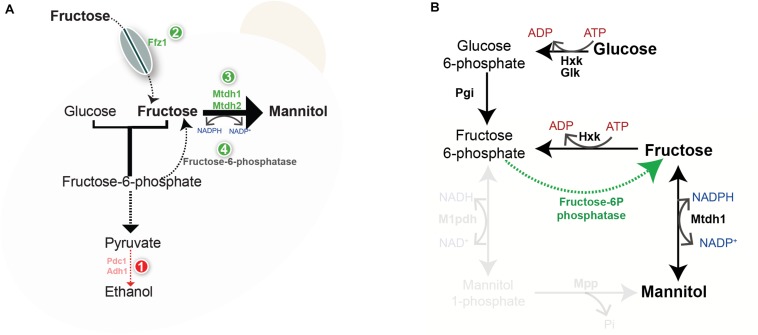
Working model for the evolution of mannitol metabolism. **(A)** Lack of or inefficient alcoholic fermentation (1) causes redox constraints that can be partly alleviated by oxidized cofactor recycling by the mannitol dehydrogenase reaction. Acquisition of Ffz1 (2) and duplication of the *MtDH* gene (3) may have supported an increase in the flux toward mannitol production. In order to harvest the advantages of mannitol production also when glucose is the sole carbon and energy source a novel pathway (4) emerged converting fructose-6-P to fructose. **(B)** Novel pathway mediating the production of mannitol from glucose in W/S-clade yeasts; Hxk- Hexokinase, Glk- Glucokinase; Pgi- Phosphoglucose isomerase, Mtdh1- Mannitol dehydrogenase. Reactions involving M1pdh and Mpp are faded to indicate that they are absent in W/S-clade species.

We propose a model (Figure 5) in which the advantage of preferential utilization of fructose would have arisen first in a background of lack of, or inefficient alcoholic fermentation, similarly to what is currently observed in fructophilic bacteria (Maeno et al., 2016, 2019). The advantage of fructose under these circumstances would be likewise to provide a means to alleviate redox imbalance by facilitating cofactor recycling through mannitol synthesis, which concomitantly oxidizes NADPH. Although alcoholic fermentation in yeasts in general oxidizes only NADH, extant W/S-clade species have alcohol dehydrogenases that operate using both NADH and NADPH as cofactors (Gonçalves et al., 2018). The likelihood of the proposed model depends on whether fructose reduction to mannitol was capable of minimizing redox balance problems resulting from loss of alcoholic fermentation. This would require either that the cofactors involved in both processes were the same in the W/S ancestor that lost alcoholic fermentation or that mechanisms existed for the equilibration of phosphorylated and unphosphorylated nicotinamide adenine cofactors. Such mechanisms have not been identified in extant yeast species (Bruinenberg et al., 1983). In this model, mannitol may have acquired later a role in stress protection in addition to its role in redox balancing. Evidence for the ancestral importance of mannitol synthesis, in addition to the evolutionary novelty of the pathway mediating glucose to fructose conversion, resides in the duplication of the mannitol dehydrogenase gene, which occurred in the W/S lineage. After efficient alcoholic fermentation was again in place, through acquisition of a bacterial gene and recruitment of the Aro10 decarboxylase for alcoholic fermentation (Gonçalves et al., 2018), the contribution of mannitol biosynthesis to redox balance may have become less important, possibly allowing one of the paralogous genes, *MtDH2*, to acquire a different main function. In conclusion, the surprisingly high flux through the novel pathway involving a fructose-6P phosphatase further underscores the link between fructophily and mannitol synthesis, since it ensures a supply of fructose even when it is not available in the environment, apparently solely for the purpose of mannitol production.

## Data Availability Statement

Publicly available datasets were analyzed in this study. This data can be found here: Bioproject PRJNA416493.

## Author Contributions

PG, HS, CG, MS-O, and LG designed the research. CG, CF, LG, and ML performed the experiments and analyzed the data. DT built the metabolic model and calculated the fluxes. PG wrote the manuscript with the assistance from HS, CG, and MS-O. DT, ML, and LG proofread the manuscript. PG and HS were responsible for funding acquisition. All authors read and approved the final manuscript.

## Conflict of Interest

The authors declare that the research was conducted in the absence of any commercial or financial relationships that could be construed as a potential conflict of interest.

## Footnotes

1 www.hmdb.ca

2 http://www.bmrb.wisc.edu/metabolomics/database
