# Supplementary material for: A New Pathway for Mannitol Metabolism in Yeasts Suggests a Link to the Evolution of Alcoholic Fermentation
Source: Front Microbiol. 2019 Nov 1;10:2510. doi: 10.3389/fmicb.2019.02510 (PMC6838020; doi:10.3389/fmicb.2019.02510)
Supplement: Supplementary file 2 [file Data_Sheet_2.docx]

**A New Pathway for Mannitol Metabolism in Yeasts Suggests a Link to the Evolution of Alcoholic Fermentation**

**Carla Gonçalves*^1^, Carolina Ferreira*^1^, Luís G. Gonçalves^2^, David L. Turner^2^, Maria José Leandro^2^, Madalena Salema-Oom^1,3^, Helena Santos^2^ and Paula Gonçalves^1^**

**SUPPLEMENTARY MATERIAL**

**Includes:**

Supplementary Figure S1 to S6

Supplementary Tables S1 to S3

Other supplementary material (Supplementary Data S1 and files referring to the phylogenetic analyses can be found in Figshare DOI: 10.6084/m9.figshare.9959693)


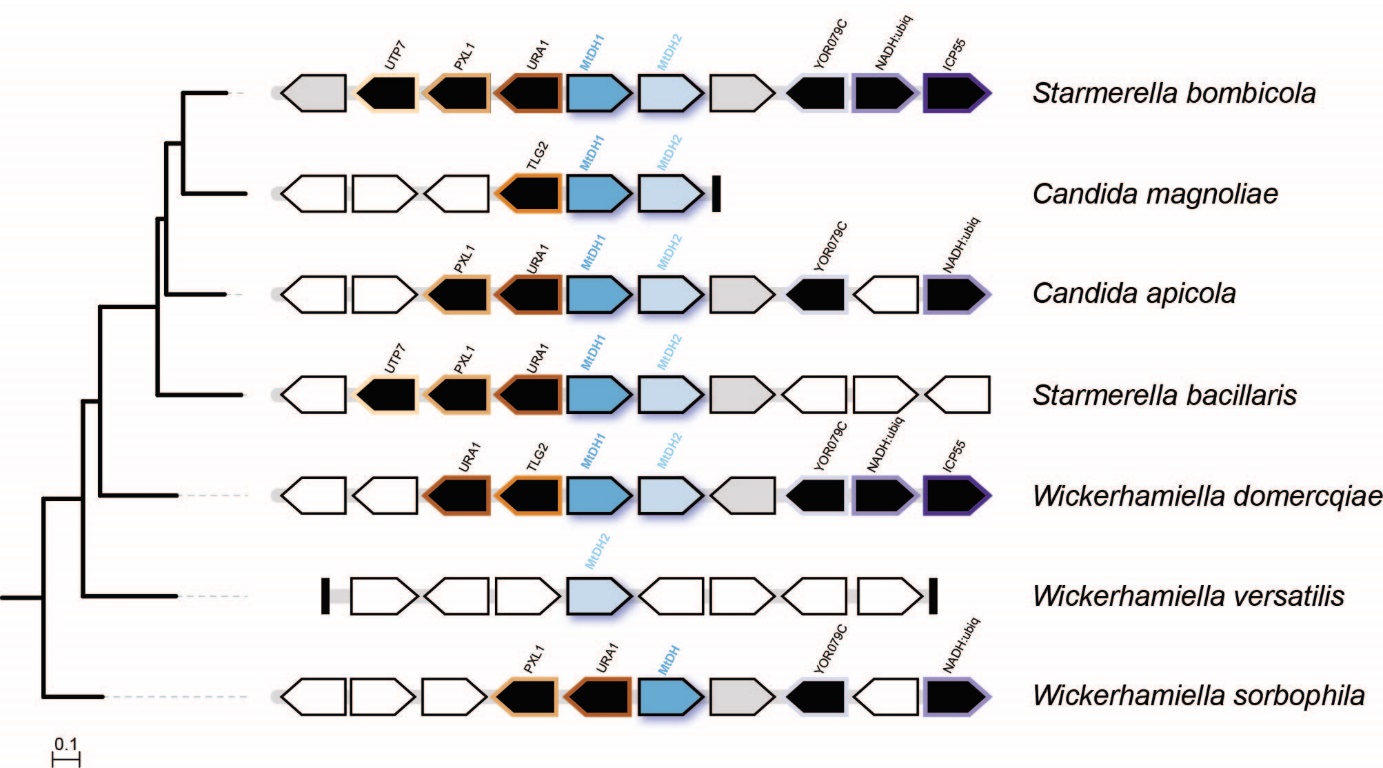


**Supplementary figure S1. Microsynteny around *MtDH* genes in the W/S clade.** Scaffolds encompassing *MtDH* genes in the species represented in the phylogeny on the left (adapted from Gonçalves C. *et al*., 2018) are depicted by grey bars. Orthologous genes are represented in black with the same stroke colour while non-syntenic genes are coloured in white. Genes encoding hypothetical proteins with no assigned function are represented in grey. For *Wickerhamiella versatilis* and *Candida magnoliae*, which encode more paralogs, only one representative scaffold is shown. In *W. versatilis*, none of the *MtDH* paralogs is syntenic with other W/S-clade species. Accession numbers for W/S-clade genomes are the following: GCA_003033765.1 (*St. bacillaris* PYCC 3044), GCA_001005415.1 (*C. apicola* NRRL Y-50540), GCA_003033435.1 (*St. bombicola* PYCC 5882), GCA_003033705.1 (C*. magnoliae* PYCC 2903), GCA_001600375.1 (*W. domercqiae* PYCC 3067), GCA_001005415.1 (*W. versatilis* JCM 5958) and NW_020193984.1 (*W. sorbophila* DS02).


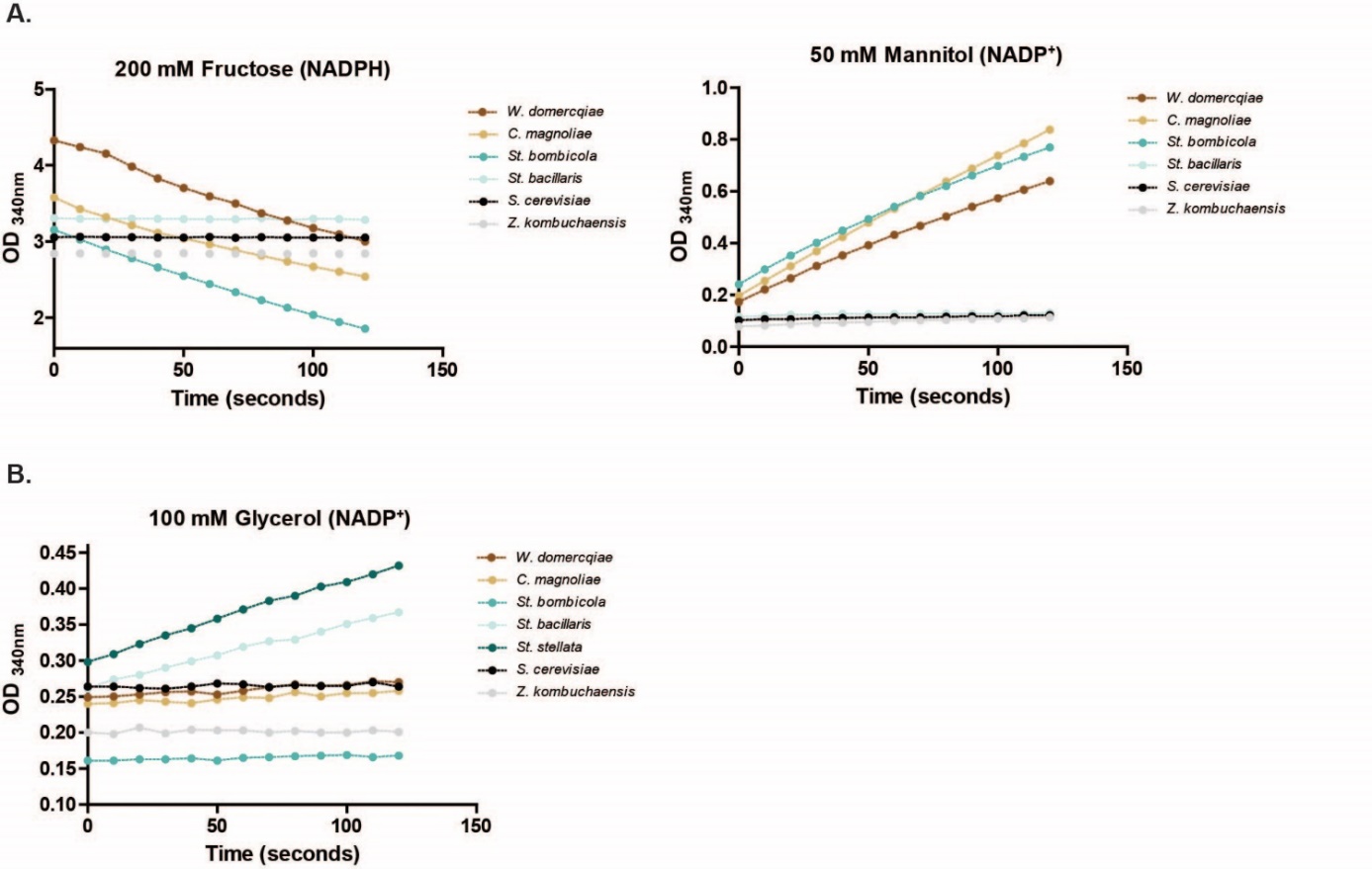


**Supplementary figure S2. (A)** Mannitol dehydrogenase (Mtdh) activities in cell free extracts of W/S species using 200 mM fructose and 50 mM mannitol as substrates and NADPH and NADP^+^ as cofactors, as indicated. **(B)** Glycerol dehydrogenase activity in cell free extracts of W/S species using 100 mM glycerol as a substrate and NADP^+^ as a cofactor. *Starmerella stellata* PYCC 2912 was used to confirm the NADP^+^-dependent glycerol dehydrogenase activity observed in the sister species *Starmerella bacillaris*. Non-fructophilic species *Saccharomyces cerevisiae* S288C and fructophilic species *Zygosaccharomyces kombuchaensis* CBS 8849 were used as negative controls.


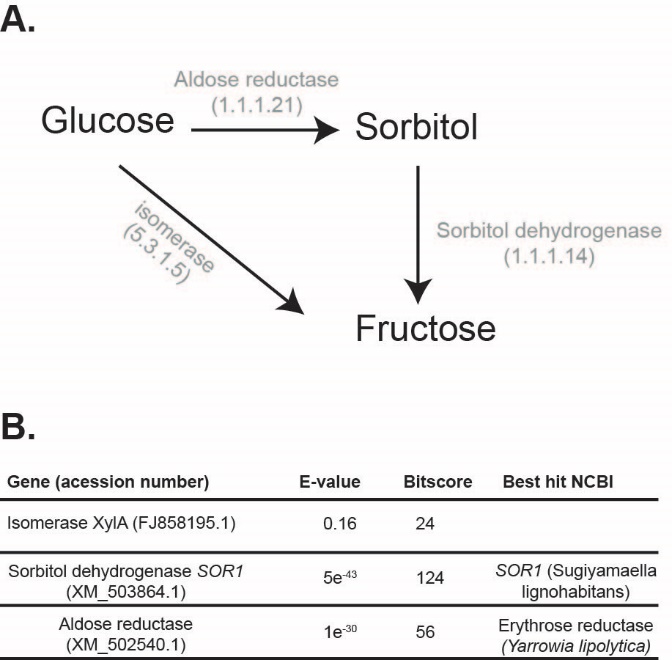


**Supplementary figure S3. Putative pathways mediating the conversion of glucose in**

**fructose. (A)** Glucose might be either converted to fructose through a glucose isomerase or through an aldose reductase/sorbitol dehydrogenase involving sorbitol as an intermediate. **(B)** Results for the tBLASTx search of the enzymes involved in the putative sorbitol route, showing gene names and Genbank accession numbers. The best hit obtained by the tBLASTx search against the *St. bombicola* PYCC 5882 genome using these genes as queries was subsequently analysed by BLASTp and the respective best hit in NCBI database is indicated (Best hit NCBI).


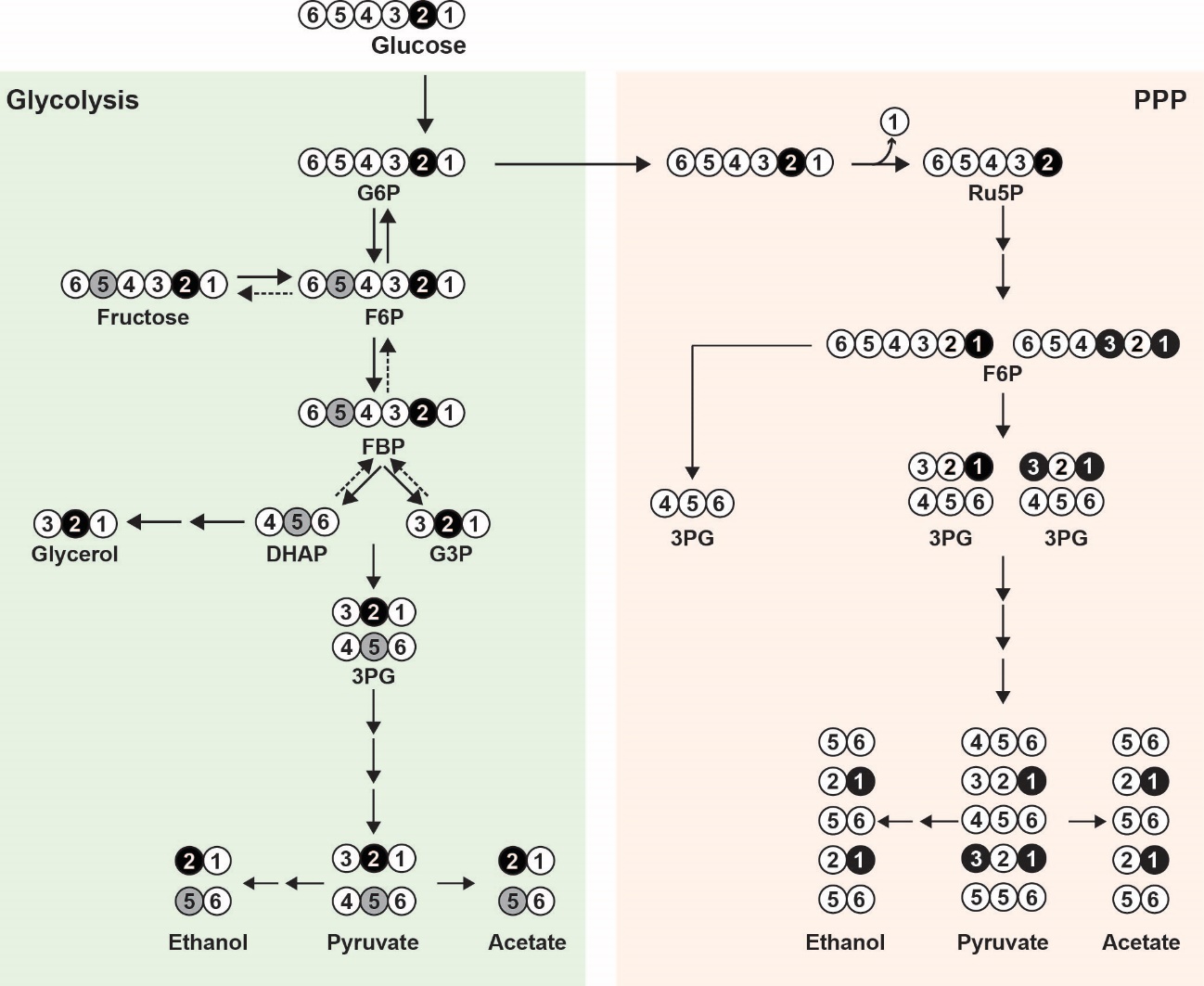


**Supplementary figure S4.** Labelling pattern of metabolites derived from [2-^13^C]glucose via the glycolytic and pentose phosphate pathways. The black circles represent the label directly originating from the [2-^13^C]glucose provided. The grey circles designate the label derived from reversed flux from the glycolytic triose phosphate pool. To avoid excessive complexity of the scheme the labelling due to reversed flux was not considered as input to the PPP. Only the first cycle of PPP is depicted.


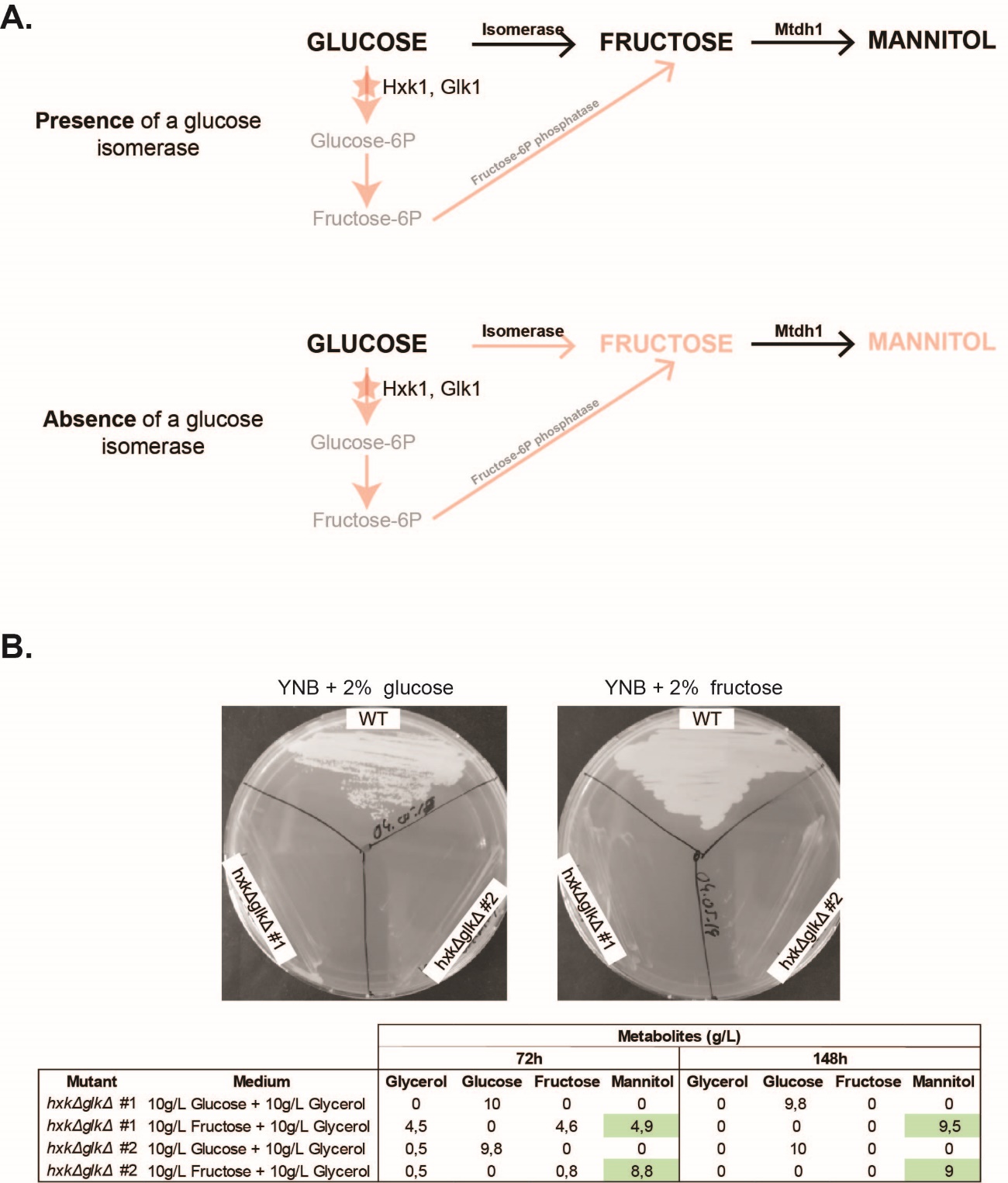


**Supplementary figure S5. Genetic evidence for the absence of a glucose isomerase in *St. bombicola* using a deletion mutant unable to phosphorylate glucose and fructose (*hxkΔglkΔ*). (A)** Expected results for the production of mannitol in the *hxkΔglkΔ* in the presence (black arrow) or absence (faded red arrow) of a glucose isomerase. In the presence of a glucose isomerase, mannitol can be produced when either glucose or fructose are provided in the growth medium. If no glucose isomerase activity is present, mannitol can only be produced when fructose is provided. (**B**) Growth phenotype of the *hxkΔglkΔ* mutant in fructose- and glucose-supplemented plates after 48 hours of incubation at 25ºC. The results for the production of mannitol in this mutant (two different transformants from two independent transformations) in YP medium supplemented with 1% (w/v) of glycerol and 1% (w/v) of glucose or 1% (w/v) of glycerol and 1% (w/v) of fructose are shown in the table. The mutants were also grown in medium containing 2% (w/v) of glycerol and no mannitol was detected which supports the conclusion that the mannitol produced by the mutants in 1% (w/v) of glycerol and 1% (w/v) of fructose comes from the available fructose. The results are in line with the absence of a glucose isomerase as no mannitol or fructose could be detected in culture supernatants when glucose was provided in the growth medium.


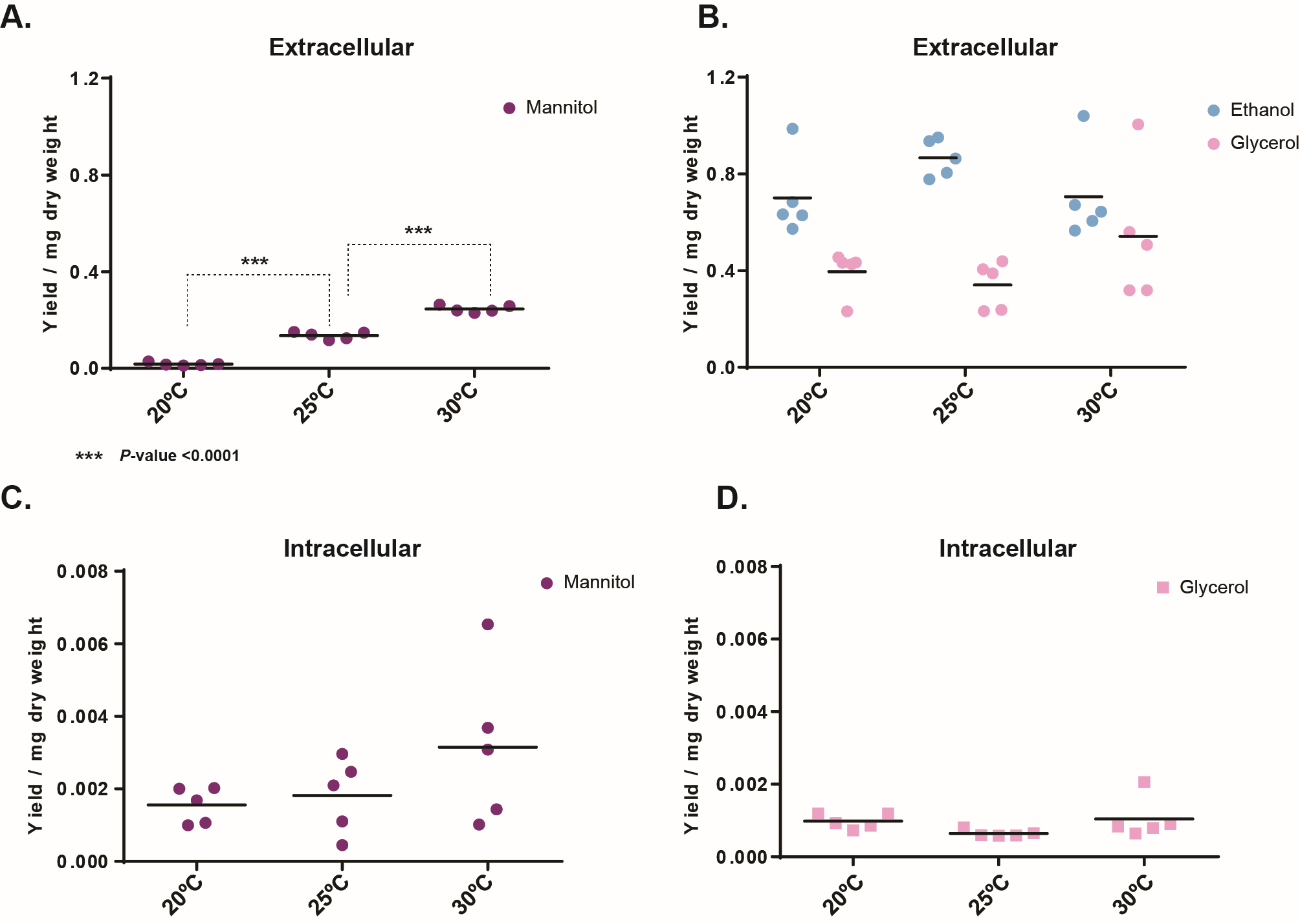


**Supplementary figure S6**. **Effect of temperature on the production of mannitol, glycerol and ethanol in *St. bombicola* wild type**. Extracellular concentration (mg/mg of sugar consumed/mg dry weight) of mannitol (A), ethanol and glycerol (B) measured in culture supernatants of *St. bombicola*. Intracellular mannitol (C) and glycerol (D) production measured in cell-free extracts of *St. bombicola* wild type at 20 ºC, 25 ºC and 30 ºC. The cells were grown at 20ºC, 25ºC and 30ºC on YP supplemented with 10% (w/v) glucose and 10% (w/v) fructose (20FG) for 72 hours. Horizontal lines represent the mean values of five independent assays. Statistically significant differences (one-way ANOVA, using the Bonferroni’s correction) between different temperatures for metabolite production are shown (*** p-value<0.001).

**Supplementary table S1**. List of primers used for the construction of *St. bombicola* *knock-out* mutants.

| **Mutants**  **Genotypes** | **Primers** | **Sequence 5’-3’** | **Description** |
| --- | --- | --- | --- |
| *mtdh1∆*  *mtdh1∆::HYG* | MDH1_SpeI_Fw | TACAGGACTAGTACAAAGTTCAGGGCACG | Amplification of *MtDH*1 gene plus ~1Kb upstream and downstream for ligation in p415TEF plasmid using the restriction enzymes *SpeI* and *KpnI*. *MtDH1* was subsequently disrupted using the restriction enzymes *SphI* and *SalI*. |
|  | MDH1_KpnI_Rv | CCTGTAGGTACCCGTTGAGAGCGATATC |  |
|  | MDH1_up_Fw | CAAAGTTCAGGGCACG | Amplification of *MtDH1* deletion cassette for *St. bombicola* transformation |
|  | MDH1_down_Rv | CCGTTGAGAGCGATATC |  |
|  |  |  |  |
| *mtdh2∆*  *mtdh2∆::HYG* | MDH2_Not1_Fw | AAGTCGGCGGCCGCCTGCTTACTTTGTAAACCTC | Amplification of *MtDH2* gene plus ~1Kb upstream and downstream for ligation in pJET 1.2 plasmid using the restriction enzymes *NotI* and *NcoI. MtDH2* gene was subsequently disrupted using the restriction enzymes *BglII* and *NheI*. |
|  | MDH2_NcoI_Rv | CGACTTCCATGGTCTTGGGCGGGTTCC |  |
|  | MDH2_Fw | CTGCTTACTTTGTAAACCTC | Amplification of *MtDH1* deletion cassette for *St. bombicola* transformation |
|  | MDH2_Rv | TCTTGGGCGGGTTCC |  |
|  |  |  |  |
| *mtdh1∆mtdh2∆ mdh1∆::HYG/ mdh2∆::HYG* | MDH12_SmaI_Fw | TACAGGCCCGGGCAAAGTTCAGGGCACG | Amplification of *MtDH* region plus ~1Kb upstream and downstream for ligation in p415TEF plasmid using the restriction enzymes *SmaI* and *KpnI*. *MtDH1* and *Mtdh2* were simultaneously disrupted using the restriction enzymes *SphI* and *NheI*. |
|  | MDH12_Rv | CACTCAGCCTGAGTTCTG |  |
|  | MDH1_up_Fw | CAAAGTTCAGGGCACG | Amplification of *MtDH1&2* deletion cassette for *St. bombicola* transformation |
|  | MDH2_Rv | TCTTGGGCGGGTTCC |  |
|  |  |  |  |
| *glk1∆*  *glk1∆::ZEO* | GLK1_ClaI_Fw | TATCTTATCGATACCATCGGCGTTCTCG | Amplification of *GLK1* gene with ~1Kb upstream and downstream for subsequent cloning into *PJET 1.2* using the restriction enzymes *ClaI* and *NotI. GLK1* gene was subsequently disrupted using the restriction enzymes *NheI* and *BglII***.** |
|  | GLK1_NotI_Rv | AAGATAGCGGCCGCTGTCTATGTTCAATGTCTATG |  |
|  | GLK1_Fw | CTTTCCGACAGTATAGTG | Amplification of the *glk1::ZEO* cassette |
|  | GLK1_Rv | TCTATGTAATTCGTGTCTATG |  |
| *hxk1∆ hxk1∆::HYG* | HXK_upKpn2I_Fw | GTGTACTGGAATGGCAG | Amplification of *HXK1* gene with ~1Kb upstream and downstream for subsequent cloning into *PJET 1.2* using the restriction enzymes *Kpn2I* and *NotI. HXK1* gene was subsequently disrupted using the restriction enzymes *SalI* and *StuI*. |
|  | HXK_NotI_Rv | TAAGTAGCGGCCGCTCGAATCACGAATTAGCAG |  |
|  | HXK_Fw | TCACAAACTTGGCAATACC | Amplification of the *hxk1::HYG* cassette |
|  | HXK_Rv | TCGAATCACGAATTAGCAG |  |

|  | ***M1PDH* (KEY81154.1)** | | ***MPP* (AIC75600.1)** | | ***MtDH* (Q6CEE9.1)** | |
| --- | --- | --- | --- | --- | --- | --- |
| **Species** | ***e-value*** | **bitscore** | ***e-value*** | **bitscore** | ***e-value*** | **bitscore** |
| *St. bombicola* | 0.60 | 31 | 0.019 | 35 | 1.02e^-85^ | 194 |
| *St. bacillaris* | 0.11 | 28 | 0.24 | 33 | 4e^-88^ | 202 |
| *C. magnoliae* | 0.003 | 39 | 6e^-4^ | 41 | 4e^-91^ | 226 |
| *W. domercqiae* | 0.057 | 35 | 0.009 | 37 | 5e^-108^ | 363 |
| *W. versatilis* | 0.018 | 36 | 0.048 | 35 | 2e^-78^ | 219 |
| *W. sorbophila* | 0.010 | 37 | 0.13 | 34 | 5e^-104^ | 350 |

**Supplementary table S2.** Results of tBLASTx searches of genes involved in mannitol biosynthesis in local genome databases of W/S-clade species. *E-value* and bitscore values are shown.

**Supplementary table S3**. Kinetics of ^13^C-labeled products derived from the metabolism of [1-^13^C]glucose or [2-^13^C]glucose by resting cell suspensions of *St. bombicola* with deletion of *MtDH1* and *MtDH2.* The numbers represent concentrations in mM. The compounds were quantified by ^13^C-NMR in supernatant solutions obtained from culture aliquots sampled at 0, 15, 30, 45, 60 and 120 minutes after glucose supply. The position of ^13^C label in each compound is abbreviated, e.g. [2-^13^C]glucose as 2-glucose.
